# Health-related quality of life in advanced gastric/gastroesophageal junction cancer with second-line pembrolizumab in KEYNOTE-061

**DOI:** 10.1007/s10120-021-01200-w

**Published:** 2021-08-07

**Authors:** Eric Van Cutsem, Mayur Amonkar, Charles S. Fuchs, Maria Alsina, Mustafa Özgüroğlu, Yung-Jue Bang, Hyun Cheol Chung, Kei Muro, Eray Goekkurt, Al B. Benson, Weijing Sun, Zev A. Wainberg, Josephine M. Norquist, Xinqun Chen, Chie-Schin Shih, Kohei Shitara

**Affiliations:** 1 Department of Digestive Oncology, University Hospitals Gasthuisberg Leuven and KU Leuven, 49 Herestraat, Leuven, Belgium; 2 Center for Observational and Real World Evidence, Merck & Co., Inc., Kenilworth, NJ USA; 3 Yale Cancer Center, Yale School of Medicine, New Haven, CT USA; 4 Department of Medical Oncology, Vall d’Hebron University Hospital and Institute of Oncology, University Autònoma de Barcelona, Barcelona, Spain; 5 Department of Internal Medicine, Medical Oncology, and Clinical Trial Unit, Cerrahpaşa School of Medicine, Istanbul University–Cerrahpaşa, Istanbul, Turkey; 6 Department of Internal Medicine, Seoul National University College of Medicine, Seoul, South Korea; 7 Department of Medical Oncology, Yonsei Cancer Center, Yonsei University College of Medicine, Seoul, South Korea; 8 Department of Clinical Oncology, Aichi Cancer Center Hospital, Nagoya, Japan; 9 North-German Trial Center for Innovative Oncology, Hematology Oncology Practice Eppendorf, University Cancer Center Hamburg, Hamburg, Germany; 10 Robert H. Lurie Comprehensive Cancer Center, Division of Hematology-Oncology, Feinberg School of Medicine, Northwestern University, Chicago, IL USA; 11 Department of Internal Medicine and Medical Oncology, University of Kansas, Westwood, KS USA; 12 Department of Medicine and Hematology and Oncology, David Geffen School of Medicine at UCLA, Los Angeles, CA USA; 13 Department of Medical Oncology, Merck & Co., Inc., Kenilworth, NJ USA; 14 Department of Gastrointestinal Oncology, National Cancer Center Hospital, Chiba, Japan

**Keywords:** Pembrolizumab, Chemotherapy, Gastric cancer, HRQoL

## Abstract

**Background:**

In the primary analysis population (i.e., PD-L1 combined positive score [CPS] ≥ 1) of the phase 3 KEYNOTE-061 study (NCT02370498), pembrolizumab did not significantly prolong overall survival or progression-free survival. Pembrolizumab had a favorable safety profile in the all-patient population. We present results of prespecified health-related quality of life (HRQoL) analyses.

**Methods:**

HRQoL was measured using the European Organisation for Research and Treatment of Cancer (EORTC) Quality of Life Questionnaire Core 30 (QLQ-C30), EORTC QLQ gastric cancer questionnaire (QLQ-STO22), and EuroQol 5-dimension, 3-level questionnaire (EQ-5D-3L). Data were analyzed from patients who received ≥ 1 dose of study treatment and who completed ≥ 1 HRQoL assessment. Key analyses included baseline to week 12 least-squares mean (LSM) change in global health status (GHS)/QoL, functional/symptom subscales, and time to deterioration (TTD; ≥ 10-point decrease from baseline) for specific subscales.

**Results:**

The HRQoL population included 371 patients (pembrolizumab, *n* = 188; paclitaxel, *n* = 183). Compliance and completion rates for all 3 questionnaires were similar in both groups at baseline and week 12. There was no difference in LSM change between groups (– 3.54; 95% CI – 8.92 to 1.84) in GHS/QoL at week 12. LSM change from baseline to week 12 for most QLQ-C30, QLQ-STO22, and EQ-5D-3L subscales indicated some worsening of QoL in both groups. TTD for GHS/QoL, nausea/vomiting, and appetite loss subscales in QLQ-C30 and the pain subscales in QLQ-STO22 were similar between treatment groups.

**Conclusions:**

In this population with advanced gastric and GEJ cancer receiving second-line treatment, HRQoL was similar in patients receiving pembrolizumab and those receiving paclitaxel.

**Clinical trial registry and number:**

ClinicalTrials.gov, NCT02370498.

**Supplementary Information:**

The online version contains supplementary material available at 10.1007/s10120-021-01200-w.

## Introduction

Gastric cancer is the fifth most common cancer worldwide; in 2018 alone, more than 1 million new cases were diagnosed globally and nearly 800,000 deaths occurred [1]. Patients with advanced-stage gastric and/or gastroesophageal junction (GEJ) cancer experience diminished health-related quality of life (HRQoL)—overall health status and functioning decrease while the burden of cancer-related symptoms increases [2, 3]. The type of symptom burden also changes; weight loss, abdominal pain, vomiting, gastric obstruction, and gastric bleeding are more commonly associated with advanced-stage gastric/GEJ [2]. In addition to disease-related symptoms, those related to chemotherapy in patients with advanced gastric/GEJ cancer include abdominal pain, fatigue, nausea/vomiting, and diarrhea [2]. Treatments for gastric/GEJ cancer should, therefore, be evaluated for their effects on patients’ already diminished HRQoL associated with the disease.

The current recommended systemic treatment options for gastric/GEJ cancer include first-line treatment with platinum plus fluoropyrimidine (in combination with trastuzumab in HER2–positive tumors) and second-line or subsequent treatment with docetaxel, paclitaxel, irinotecan, fluorouracil and irinotecan, or paclitaxel and ramucirumab or with pembrolizumab in patients with high microsatellite instability or mismatch protein repair–deficient tumors [4]. Regimens preferred in the third-line or later setting include trifluridine plus tipiracil and pembrolizumab as described below [4]. Modest improvements in HRQoL were observed for a minority of treatment regimens in the first-line setting—single-agent chemotherapy led to clinically significant improvement in overall, function, and symptom scores over approximately 4 months. In the second-line or later setting, overall HRQoL remained stable for nearly all treatments, whereas role functioning, fatigue, appetite loss, and distress from hair loss scores worsened over time [5].

Pembrolizumab, an anti–programmed death 1 (PD-1) monoclonal antibody, has demonstrated antitumor activity, greater durability of response, and manageable safety in patients with advanced gastric/GEJ cancer [6, 7]. Pembrolizumab is approved by the US Food and Drug Administration as third-line or later therapy for gastric/GEJ cancer that is PD-1 ligand 1 (PD-L1)–positive (combined positive score [CPS] ≥ 1) [8]. In KEYNOTE-061 (NCT02370498), pembrolizumab did not prolong overall survival (OS) or progression-free survival as second-line therapy for advanced gastric/GEJ cancer in the primary analysis population (PD-L1 CPS ≥ 1); however, the antitumor activity of pembrolizumab (objective response rate [ORR], 16%) was more durable (median duration of response [DOR], 18.0 months) than paclitaxel (ORR, 14%; median DOR, 5.2 months) [9]. Pembrolizumab also had a better safety profile than paclitaxel. We report the results of prespecified exploratory end points for HRQoL from KEYNOTE-061.

## Methods

### Study design and patients

The methods and primary results of the KEYNOTE-061 study have been described in detail elsewhere [9]. Brief details are provided in the Online Resource Methods.

The study protocol and all amendments were approved by the institutional review board or ethics committee at each institution. The study was conducted in accordance with the protocol and its amendments and with Good Clinical Practice guidelines. All patients provided written informed consent before enrollment.

### HRQoL outcomes

The prespecified HRQoL-based exploratory objectives were to evaluate mean score changes from baseline to week 12 in HRQoL using the European Organisation for Research and Treatment of Cancer (EORTC) Quality of Life Questionnaire Core 30 (QLQ-C30) [10–13] and the EORTC QLQ gastric cancer questionnaire (QLQ-STO22) [14, 15] from baseline to week 12 and to characterize utilities using the EuroQol 5-dimension, 3-level questionnaire (EQ-5D-3L) [16, 17] among patients treated with pembrolizumab versus paclitaxel.

Other measures included completion and compliance with EORTC QLQ-C30, EORTC QLQ-STO22, and the EQ-5D-3L; mean score change from baseline to week 12 in the EORTC QLQ-C30 global health status/quality of life (GHS/QoL) scale; mean score change from baseline to week 12 for subscales and items with EORTC QLQ-C30 (5 functional dimensions: physical, role, emotional, cognitive, and social; 3 symptom scales: fatigue, nausea/vomiting, and pain; 6 single-item measures: dyspnea, sleep disturbance, appetite loss, constipation, diarrhea, and financial difficulties) and EORTC QLQ-STO22 (22 items; 5-multi-items scales [dysphagia, pain, reflux symptoms eating restrictions anxiety] and 4 single items [dry mouth, taste, body image, and hair loss]; mean score change from baseline to week 12 for EQ-5D-3L visual analog scale (VAS) and utility score; and time to deterioration (TTD) in the GHS/QoL score from baseline with EORTC QLQ-C30 and EORTC QLQ-STO22. Changes in the EORTC QLQ-C30 and EORTC QLQ-STO22 subscales and items were defined as follows: improved, ≥ 10-point increase; remained stable, no change, or change of < 10 points; deteriorated, ≤ 10-point decrease.

The HRQoL questionnaires were administered electronically by trained personnel and were completed by patients in the following order: EQ-5D-3L, EORTC QLQ-C30, and EORTC QLQ-STO22. Each questionnaire was administered at baseline and at cycles 1, 2, 3, 4, 5, 7, and 9; every 6 weeks after week 24 through 1 year or end of treatment; and at the 30-day posttreatment discontinuation follow-up visit. Study sites were instructed to make every effort to ensure that administration of HRQoL questionnaires occurred before all other study procedures.

### Statistical analysis

In this exploratory analysis with no formal hypothesis testing, data were analyzed for all patients in the primary analysis population who received ≥ 1 dose of study treatment and who completed ≥ 1 HRQoL questionnaire. Compliance and completion were summarized by treatment group and visit. Compliance was defined by the proportion of patients who completed ≥ 1 HRQoL assessment among those expected to complete the questionnaires at each visit (excluding patients missing by design because they discontinued study treatment). Completion was defined by the proportion of patients who completed ≥ 1 HRQoL assessment among the total HRQoL analysis population at each visit. The protocol-specified primary HRQoL end point was least-squares mean (LSM) change from baseline to week 12, which was assessed using a constrained longitudinal data analysis model, with HRQoL score as the response variable and treatment-by-time interaction and trial stratification factors as covariates.

Multiple imputations based on the missing-at-random assumption were used in the analysis of improved, stable, and deteriorated results from baseline to week 12. TTD in EORTC QLQ-C30 GHS/QoL score was estimated using the Kaplan–Meier method; the hazard ratio was estimated with a stratified (by geographic region and time to progression on first-line therapy) Cox proportional hazards model.

## Results

### Patients

The primary study population comprised 395 patients with CPS ≥ 1 (pembrolizumab, *n* = 196; paclitaxel, *n* = 199) [9]. At data cutoff (October 26, 2017), 2 patients in the pembrolizumab group and 11 in the paclitaxel group had not received study medication and 6 and 5 patients, respectively, had not completed an HRQoL questionnaire. The HRQoL analysis population therefore included 188 patients in the pembrolizumab group and 183 patients in the paclitaxel group.

### HRQoL compliance and completion

EORTC QLQ-C30 compliance rates were 92.0% and 92.9%, respectively, at baseline in the pembrolizumab group and the paclitaxel group and remained high (86.6% and 82.1%, respectively) for patients on study at week 12 (Table 1). Completion rates decreased over time, and 51.6% of patients in the pembrolizumab group and 55.2% of patients in the paclitaxel group completed the questionnaire at week 12. At week 12, patients in the pembrolizumab group discontinued because of disease progression (31.4%), patient/physician decision (2.2%), adverse event (AE; 1.1%), or death (1.1%) and patients in the paclitaxel group discontinued because of disease progression (22.4%), patient/physician decision (3.3%), AE (4.9%), or death (0.5%). EORTC QLQ-STO22 and EQ-5D-3L compliance and completion rates were similar to those observed for the EORTC QLQ-C30 (Online Resource Tables 1 and 2).

**Table 1:** Rates of compliance and completion of the EORTC QLQ-C30

Treatment Visit	Compliance^a^		Completion^b^	
	Pembrolizumab *n*/*N* (%)	Paclitaxel *n*/*N* (%)	Pembrolizumab *n*/*N* (%)	Paclitaxel *n*/*N* (%)
Baseline	173/188 (92.0)	170/183 (92.9)	173/188 (92.0)	170/183 (92.9)
Week 3 or 4	161/178 (90.4)	127/179 (70.9)	161/188 (85.6)	127/183 (69.4)
Week 6	134/159 (84.3)	117/162 (72.2)	134/188 (71.3)	117/183 (63.9)
Week 9	113/132 (85.6)	107/140 (76.4)	113/188 (60.1)	107/183 (58.5)
Week 12	97/112 (86.6)	101/123 (82.1)	97/188 (51.6)	101/183 (55.2)
Week 18	77/91 (84.6)	70/95 (73.7)	77/188 (41.0)	70/183 (38.3)
Week 24	50/69 (73.5)	42/62 (67.7)	50/188 (26.6)	42/183 (23.0)
Week 30	44/60 (73.3)	18/32 (56.3)	44/188 (23.4)	18/183 (9.8)
Week 36	35/48 (72.9)	12/20 (60.0)	35/188 (18.6)	12/183 (6.6)
Week 42	27/39 (69.2)	11/17 (64.7)	27/188 (14.4)	11/183 (6.0)
Week 48	27/36 (75.0)	7/9 (77.8)	27/188 (14.4)	7/183 (3.8)

*EORTC* European Organisation for Research and Treatment of Cancer, *QLQ-C30* Quality of Life Questionnaire Core 30

^a^The proportion of patients who completed ≥ 1 HRQoL assessment among those expected to complete the instruments at each visit, excluding those missing by design

^b^The proportion of patients who completed ≥ 1 HRQoL assessment among the total HRQoL analysis population at each visit

**Table 2:** Change from baseline to week 12 in EORTC QLQ-C30 GHS/QOL scores

Results	Pembrolizumab *n* = 188	Paclitaxel *n* = 183
Baseline		
* n*	173	171
Mean (SD)	63.05 (21.326)	62.57 (20.953)
Week 12		
* n*^a^	98	106
Mean (SD)	63.27 (22.918)	63.68 (20.483)
Change from baseline to week 12, LSM (95% CI)	− 6.84 (− 10.87 to − 2.81)	− 3.30 (− 7.22 to 0.61)
Difference in LSM (95% CI)	− 3.54 (− 8.92 to 1.84)	
*p* value	0.196	

*EORTC* European Organisation for Research and Treatment of Cancer, *LSM* least-squares mean, *QLQ-C30* Quality of Life Questionnaire Core 30, *SD* standard deviation

^a^Number of patients in each treatment group with nonmissing assessments at this time point

### Change from baseline in HRQoL

#### EORTC QLQ-C30

**Fig. 1:**
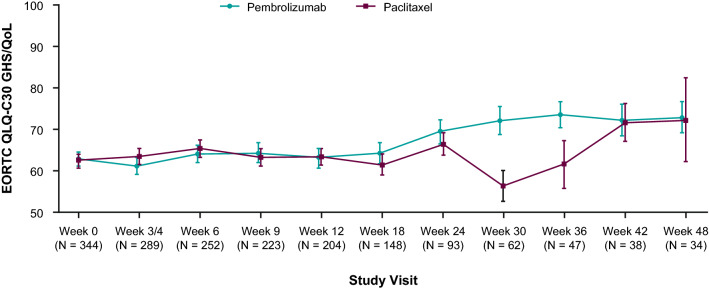
Mean (SE) EORTC QLQ-C30 GHS/QoL scores over time. *EORTC* European Organisation for Research and Treatment of Cancer, *GHS* global health status, *Q3W* every 3 weeks, *QLQ-30* Quality of Life Questionnaire Core 30, *QoL* quality of life, *SE* standard error

**Fig. 2:**
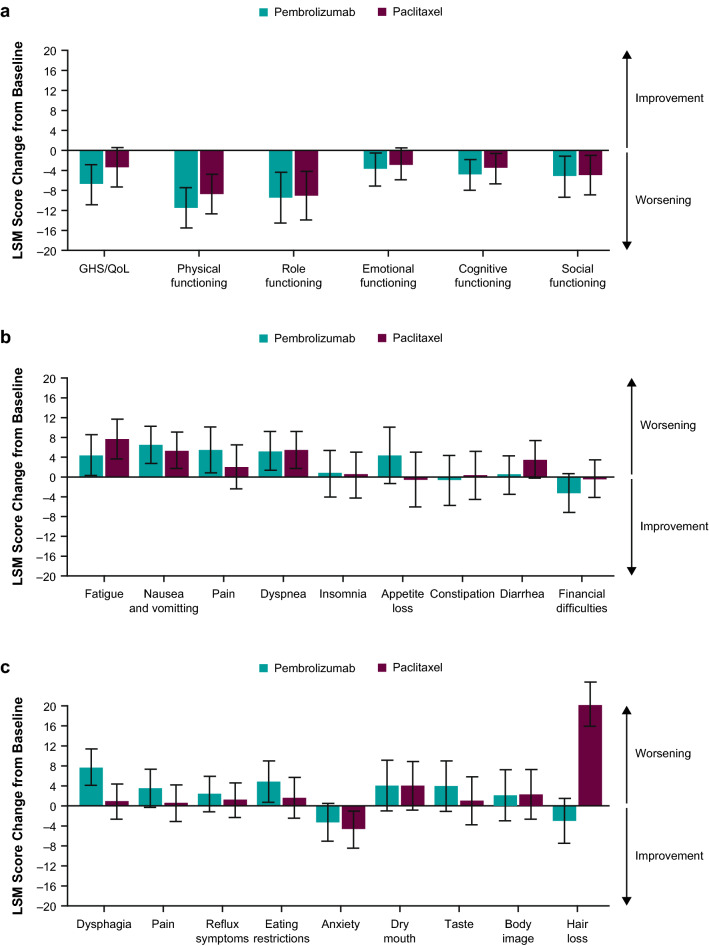
LSM (95% CI) change from baseline to week 12 in **a** EORTC QLQ-C30 GHS/QoL and functional subscale scores, **b** EORTC QLQ-C30 symptom subscale scores, and **c** EORTC QLQ-STO22 symptom subscale scores. *EORTC* European Organisation for Research and Treatment of Cancer, *GHS* global health status, *LSM* least-squares mean, *Q3W* every 3 weeks, *QLQ-30* Quality of Life Questionnaire Core 30, *QLQ-STO22* health-related QoL questionnaire in gastric cancer, *QoL* quality of life

Baseline GHS/QoL mean scores were similar between treatment groups (pembrolizumab, 63.05 [SD, 21.326]; paclitaxel, 62.57 [SD, 20.953]) (Table 2). GHS/QoL mean scores worsened slightly from baseline to week 12 in both treatment groups (LSM change: − 6.84 for pembrolizumab [95% CI –10.87 to –2.81], – 3.30 for paclitaxel [95% CI – 7.22 to 0.61]) (Fig. 1). The LSM score change from baseline to week 12 in GHS/QoL score was slightly worse in the pembrolizumab group versus the paclitaxel group (difference, –3.54; 95% CI – 8.92 to 1.84; nominal *p* = 0.196). At week 12, LSM score changes from baseline indicated worsening of all EORTC QLQ-C30 functional subscale and of most symptom subscale scores in both treatment groups (Fig. 2a, b). Worsening in these domains was similar in magnitude between pembrolizumab and paclitaxel (Fig. 2a, b). Beginning at week 18, GHS/QoL mean scores showed improvement for patients receiving pembrolizumab (Fig. 1). This trend was not observed in the paclitaxel group; however, comparison of GHS/QoL mean scores for patients receiving paclitaxel is limited because of low completion rates beginning at week 24.

#### EORTC QLQ-STO22

LSM score changes from baseline to week 12 showed worsening of most EORTC QLQ-STO22 symptom subscales for both treatment groups (Fig. 2c); only mean score changes for anxiety showed improvement in both groups. Mean score change for hair loss was nominally different between treatment groups; patients receiving paclitaxel experienced hair loss, whereas those receiving pembrolizumab did not. Mean score changes for dysphasia and eating restrictions were worse from baseline to week 12 for patients in the pembrolizumab group, but the difference was minimal compared with scores for patients in the paclitaxel group.

#### EQ-5D-3L

At week 12, LSM changes from baseline in EQ-5D-3L utility scores and VAS indicated worsening in both groups (Online Resource Fig. 1). LSM for utility scores differed between groups (difference, − 0.07; 95% CI − 0.13 to − 0.01; nominal *p* = 0.029) but not for VAS (difference − 2.37; 95% CI − 7.17 to 2.43; nominal *p* = 0.331).

### Proportion of patients with deteriorated or improved status at week 12

**Fig. 3:**
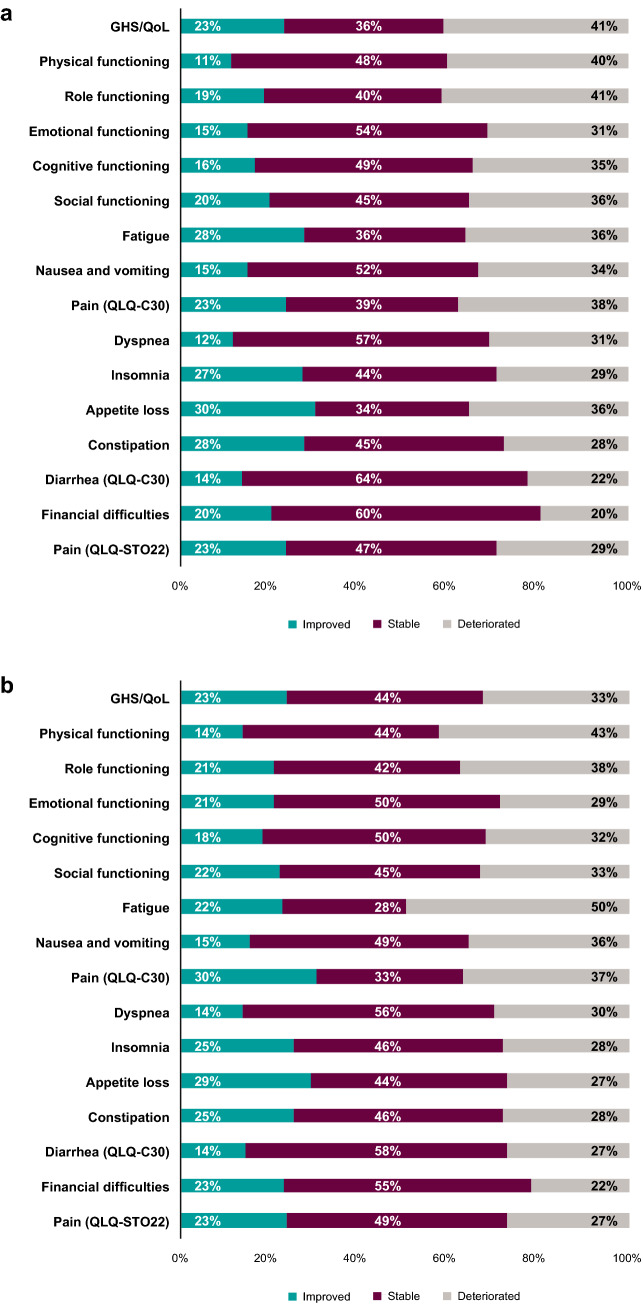
Proportion of patients with deteriorated or improved status in EORTC QLQ-C30 scores at week 12. **a** Pembrolizumab. **b** Paclitaxel. *EORTC* European Organisation for Research and Treatment of Cancer, GHS, global health status; *QLQ-30* Quality of Life Questionnaire Core 30, QLQ-STO22, health-related QoL questionnaire in gastric cancer; QoL, quality of life

At week 12, 41% and 33% of patients in the pembrolizumab group and the paclitaxel group, respectively, experienced deterioration from baseline in the EORTC QLQ-C30 GHS/QoL mean score change; 23% in both groups experienced improvement (Fig. 3). Except for fatigue, scores for functioning and symptom subscales showed similar degrees of worsening and improvement. Fatigue symptom subscale scores improved in more and deteriorated in fewer pembrolizumab-treated patients than paclitaxel-treated patients (improved, 28% pembrolizumab and 22% paclitaxel; deteriorated, 36% pembrolizumab and 50% paclitaxel) (Fig. 3).

### Time to deterioration

**Fig. 4:**
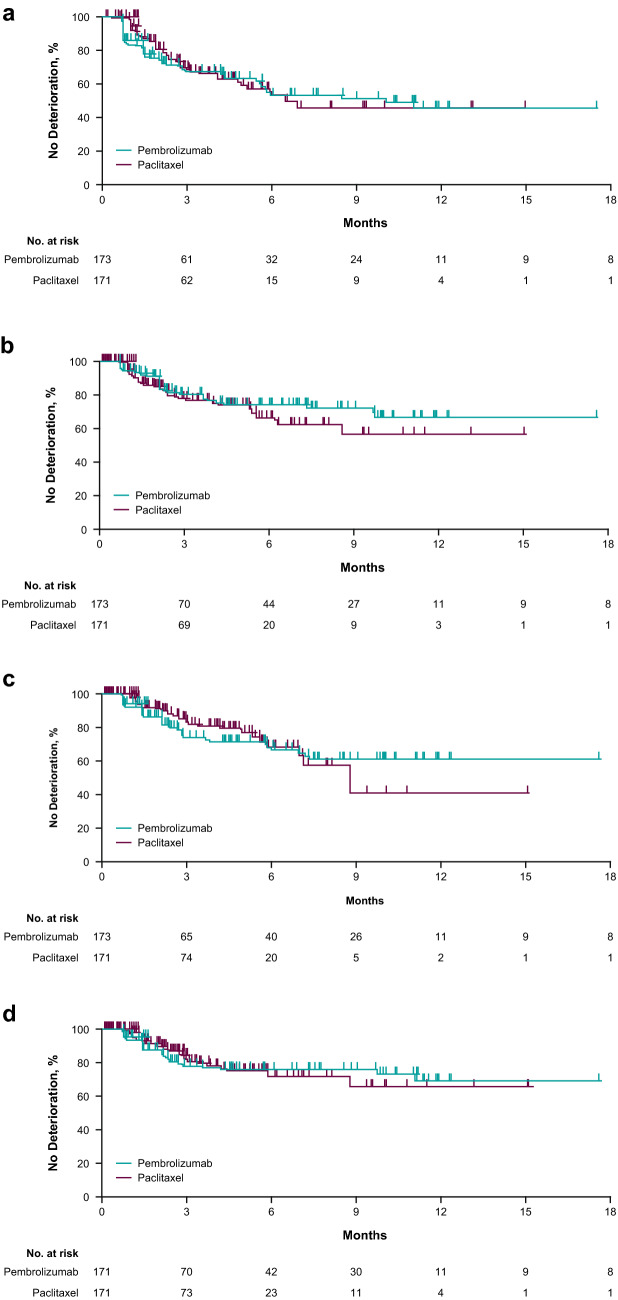
TTD in the **a** EORTC QLQ-C30 GHS/QoL scale, **b** EORTC QLQ-C30 nausea/vomiting subscale, **c** EORTC QLQ-C30 appetite loss subscale, and **d** EORTC QLQ-STO22 pain subscale. *GHS* global health status, *Q3W* every 3 weeks, *QLQ-30* Quality of Life Questionnaire Core 30, *QLQ-STO22* health-related QoL questionnaire in gastric cancer, *QoL* quality of life, *TTD* time to deterioration

Among the 41% pembrolizumab-treated and 33% of paclitaxel-treated patients who experienced deterioration in GHS/QoL scores, median TTD was 10.1 months in the pembrolizumab group and 6.9 months in the paclitaxel group (HR 1.06; 95% CI 0.71–1.58) (Fig. 4a); the Kaplan–Meier plot revealed a slightly lower curve in the first 3 months in the pembrolizumab group compared with the paclitaxel group. Median TTD was similar for pembrolizumab and paclitaxel for the EORTC QLQ-C30 subscales for nausea/vomiting (HR 0.81; 95% CI 0.50–1.33; 15% of all patients in both groups experienced deterioration) and appetite loss (HR 1.22; 95% CI 0.76–1.96; 36% of pembrolizumab-treated and 27% of paclitaxel-treated patients experienced deterioration) and for EORTC QLQ-STO22 pain (HR 1.09; 95% CI 0.65–1.83; 29% of pembrolizumab-treated and 27% of paclitaxel-treated patients experienced deterioration) (Fig. 4b–d).

## Discussion

In this prespecified exploratory analysis, the observed effects in HRQoL were comparable for patients with advanced gastric/GEJ cancer and PD-L1 CPS ≥ 1 regardless of whether they received second-line pembrolizumab monotherapy or paclitaxel. Compliance rates were high in both groups. Both treatment groups showed some worsening of functional and symptom subscale scores at week 12. The LSM of GHS/QoL scores worsened during the first 12 weeks in both treatment groups, with a confidence interval crossing zero for the difference in LSM; the LSM improved in pembrolizumab-treated patients after week 18. TTD was also similar for both groups.

Overall, HRQoL as measured by the GHS/QoL and functional subscales from baseline to week 12 worsened in both groups, with overlapping confidence intervals between the treatment groups for many of the subscale scores (Figs. 1, 2b). Nearly all symptoms measured by the EORTC QLQ-C30 and the EORTC QLQ-STO22 worsened from baseline to week 12 in both treatment groups, with many overlapping confidence intervals between the treatment groups (Fig. 2b, c). Although fatigue and diarrhea subsided in more and grew in fewer pembrolizumab-treated patients than paclitaxel-treated patients, the only symptom showing an apparent difference was hair loss, which, as expected, was worse with paclitaxel than with pembrolizumab. As with GHS/QoL scores, there was no difference in TTD at week 12 between treatment groups in the EORTC QLQ-C30 subscales for nausea/vomiting and appetite loss or in the EORTC QLQ-STO22 scale for pain. However, these results were consistent with the primary safety analysis of all patients (regardless of CPS status) from KEYNOTE-061, in which fewer patients experienced treatment-related AEs in the pembrolizumab group (53% overall, 14% grade 3–5) than in the paclitaxel group (84% and 35%, respectively) [9]. Additionally, we observed a worsening of utility scores in both treatment groups using the descriptive EQ-5D-3L questionnaire.

Interpretation of the observations from this exploratory analysis may be limited by the open-label nature of the trial and the short duration of follow-up attributed to the high rate of discontinuation, usually because of disease progression. As previously discussed in the primary analysis of KEYNOTE-061, we observed crossing of the survival curves in the second-line setting, suggesting that some pembrolizumab-treated patients experienced early disease progression and poor outcomes [9]. However, there was a trend toward better outcomes for pembrolizumab-treated patients who achieved disease control and could be maintained on therapy, as evidenced by the latter part of the survival curve [9]. These findings may be attributed to the time it takes to induce an antitumor immune response [9]. As a result, the HRQoL data must be carefully interpreted with these findings in mind. Another limitation of this HRQoL analysis was the week 12 end point because separation between the treatment groups in the GHS/QoL score appeared after that point.

## Conclusions

In the KEYNOTE-061 trial comparing pembrolizumab and paclitaxel as second-line treatments for patients with advanced gastric/GEJ cancer and PD-L1 CPS ≥ 1, the study groups experienced generally similar HRQoL from baseline through 12 weeks. In overall scores and in specific subscales and items, pembrolizumab did not appear to worsen HRQoL to a greater extent than paclitaxel. Even though the HRQoL questionnaires were not intended to evaluate AEs, these results suggest that patient-reported impacts of specific symptoms (e.g., fatigue, hair loss, decreased appetite) were consistent with the safety results of KEYNOTE-061, which showed fewer AEs with pembrolizumab than paclitaxel.

## Data sharing

Merck Sharp & Dohme Corp., a subsidiary of Merck & Co., Inc., Kenilworth, NJ, USA (MSD) is committed to providing qualified scientific researchers access to anonymized data and clinical study reports from the company’s clinical trials for the purpose of conducting legitimate scientific research. MSD is also obligated to protect the rights and privacy of trial participants and, as such, has a procedure in place for evaluating and fulfilling requests for sharing company clinical trial data with qualified external scientific researchers. The MSD data-sharing website (available at: http://engagezone.msd.com/ds_documentation.php) outlines the process and requirements for submitting a data request. Applications will be promptly assessed for completeness and policy compliance. Feasible requests will be reviewed by a committee of MSD subject matter experts to assess the scientific validity of the request and the qualifications of the requestors. In line with data privacy legislation, submitters of approved requests must enter into a standard data-sharing agreement with MSD before data access is granted. Data will be made available for request after product approval in the US and EU or after product development is discontinued. There are circumstances that may prevent MSD from sharing requested data, including country or region-specific regulations. If the request is declined, it will be communicated to the investigator. Access to genetic or exploratory biomarker data requires a detailed, hypothesis-driven statistical analysis plan that is collaboratively developed by the requestor and MSD subject matter experts; after approval of the statistical analysis plan and execution of a data-sharing agreement, MSD will either perform the proposed analyses and share the results with the requestor or will construct biomarker covariates and add them to a file with clinical data that is uploaded to an analysis portal so that the requestor can perform the proposed analyses.

## Supplementary Information

Below is the link to the electronic supplementary material.

Supplementary file1 (DOCX 115 kb)
