# Supplementary material for: Health-related quality of life in advanced gastric/gastroesophageal junction cancer with second-line pembrolizumab in KEYNOTE-061
Source: Gastric Cancer. 2021 Aug 7;24(6):1330–40. doi: 10.1007/s10120-021-01200-w (PMC8502140; doi:10.1007/s10120-021-01200-w)

***Gastric Cancer***

**Health-related quality of life in advanced gastric/gastroesophageal junction cancer with second-line pembrolizumab in KEYNOTE-061**

Eric Van Cutsem, Mayur Amonkar, Charles S. Fuchs, Maria Alsina, Mustafa Özgüroğlu, Yung-Jue Bang, Hyun Cheol Chung, Kei Muro, Eray Goekkurt, Al Benson, Weijing Sun, Zev A. Wainberg, Josephine M. Norquist, Xinqun Chen, Chie-Schin Shih, Kohei Shitara

Corresponding author: Eric Van Cutsem, Department of Digestive Oncology, University Hospitals Gasthuisberg Leuven and KU Leuven, Leuven, Belgium

**Online Resource Methods**

Briefly, KEYNOTE-061 was a randomized, multicenter, open-label, phase 3 trial in patients with advanced gastric/gastroesophageal junction (GEJ) cancer conducted to evaluate pembrolizumab 200 mg every 3 weeks for up to 35 cycles (~2 years) compared with paclitaxel 80 mg/m^2^ on days 1, 8, and 15 of each 4-week cycle. Patients could be enrolled if they were 18 years of age or older, had confirmed adenocarcinoma of the stomach or GEJ that was metastatic or locally advanced but unresectable, and had progressive disease per RECIST v1.1 after first-line platinum- and fluoropyrimidine-containing therapy. Key exclusion criteria included previous anti–PD-1, anti–PD-L1, or anti–PD-L2 therapy and active autoimmune disease necessitating systemic therapy. Randomization was assigned in a 1:1 ratio, and patients were stratified by geographic region (Europe/Israel/North America/Australia vs Asia vs rest of world), time to progression on first-line therapy (<6 months vs ≥6 months), and PD-L1 expression status (combined positive score [CPS] <1 vs CPS ≥1).

PD-L1 expression was assessed in archival or newly collected tumor samples at a central laboratory using the PD-L1 IHC 22C3 pharmDx assay (Agilent Technologies, Carpinteria, CA, USA) and measured using CPS, defined as the number of PD-L1–positive cells (tumor cells, lymphocytes, macrophages) as a proportion of the total number of tumor cells, multiplied by 100. The primary end points were overall survival and progression-free survival among patients with PD-L1 CPS ≥1 tumors.

**Online Resource Table 1**  Rates of compliance and completion of the EORTC QLQ-STO22

|  | Compliance* | | Completion^†^ | |
| --- | --- | --- | --- | --- |
|  | Pembrolizumab  *n*/*N* (%) | Paclitaxel  *n*/*N* (%) | Pembrolizumab  *n*/*N* (%) | Paclitaxel  *n*/*N* (%) |
| Baseline | 171/188 (91.0) | 170/183 (92.9) | 171/188 (91.0) | 170/183 (92.9) |
| Week 3 or 4 | 159/178 (89.3) | 127/179 (70.9) | 159/188 (84.6) | 127/183 (69.4) |
| Week 6 | 133/159 (83.6) | 116/162 (71.6) | 133/188 (70.7) | 116/183 (63.4) |
| Week 9 | 114/132 (86.4) | 107/140 (76.4) | 114/188 (60.4) | 107/183 (58.5) |
| Week 12 | 97/112 (86.6) | 101/123 (82.1) | 97/188 (51.6) | 101/183 (55.2) |
| Week 18 | 77/91 (84.6) | 69/95 (72.6) | 77/188 (41.0) | 69/183 (37.7) |
| Week 24 | 50/68 (73.5) | 42/62 (67.7) | 50/188 (26.6) | 42/183 (23.0) |
| Week 30 | 44/60 (73.3) | 18/32 (56.3) | 44/188 (23.4) | 18/183 (9.8) |
| Week 36 | 35/48 (72.9) | 12/20 (60.0) | 35/188 (18.6) | 12/183 (6.6) |
| Week 42 | 27/39 (69.2) | 11/17 (64.7) | 27/188 (14.4) | 11/183 (6.0) |
| Week 48 | 27/36 (75.0) | 7/9 (77.8) | 27/188 (14.4) | 7/183 (3.8) |

EORTC, European Organisation for Research and Treatment of Cancer; QLQ, Quality of Life Questionnaire; STO22, gastric cancer questionnaire.

*The proportion of patients who completed ≥1 HRQoL assessment among those expected to complete the instruments at each visit, excluding those missing by design.

^†^The proportion of patients who completed ≥1 HRQoL assessment among the total HRQoL analysis population at each visit.

**Online Resource Table 2** Rates of compliance and completion of the EQ-5D-3L

|  | Compliance* | | Completion^†^ | |
| --- | --- | --- | --- | --- |
|  | Pembrolizumab  *n*/*N* (%) | Paclitaxel  *n*/*N* (%) | Pembrolizumab  *n*/*N* (%) | Paclitaxel  *n*/*N* (%) |
| Baseline | 175/188 (93.1) | 171/183 (93.4) | 175/188 (93.1) | 171/183 (93.4) |
| Week 3 or 4 | 162/178 (91.0) | 126/179 (70.4) | 162/188 (86.2) | 126/183 (68.9) |
| Week 6 | 134/159 (84.3) | 117/162 (72.2) | 134/188 (71.3) | 117/183 (63.9) |
| Week 9 | 113/132 (85.6) | 107/140 (76.4) | 113/188 (60.1) | 107/183 (58.5) |
| Week 12 | 97/112 (86.6) | 101/123 (82.1) | 97/188 (51.6) | 101/183 (55.2) |
| Week 18 | 77/91 (84.6) | 70/95 (73.7) | 77/188 (41.0) | 70/183 (38.3) |
| Week 24 | 51/69 (73.9) | 43/62 (69.4) | 51/188 (27.1) | 43/183 (23.5) |
| Week 30 | 44/60 (73.3) | 18/32 (56.3) | 44/188 (23.4) | 18/183 (9.8) |
| Week 36 | 35/47 (74.5) | 12/20 (60.0) | 35/188 (18.6) | 12/183 (6.6) |
| Week 42 | 27/39 (69.2) | 11/17 (64.7) | 27/188 (14.4) | 11/183 (6.0) |
| Week 48 | 27/36 (75.0) | 7/9 (77.8) | 27/188 (14.4) | 7/183 (3.8) |

EQ-5D-3L, EuroQol 5-dimension, 3-level questionnaire.

*The proportion of patients who completed ≥1 HRQoL assessment among those expected to complete the instruments at each visit, excluding those missing by design.

^†^The proportion of patients who completed ≥1 HRQoL assessment among the total HRQoL analysis population at each visit.

**Online Resource Figure 1.** LSM (95% CI) change from baseline to week 12 in EQ-5D-3L. (A) Utility score. (B) VAS. EQ-5D-3L, EuroQol 5-dimension, 3-level questionnaire; GHS, global health status; LSM, least-squares mean; QoL, quality of life; VAS, visual analog scale.


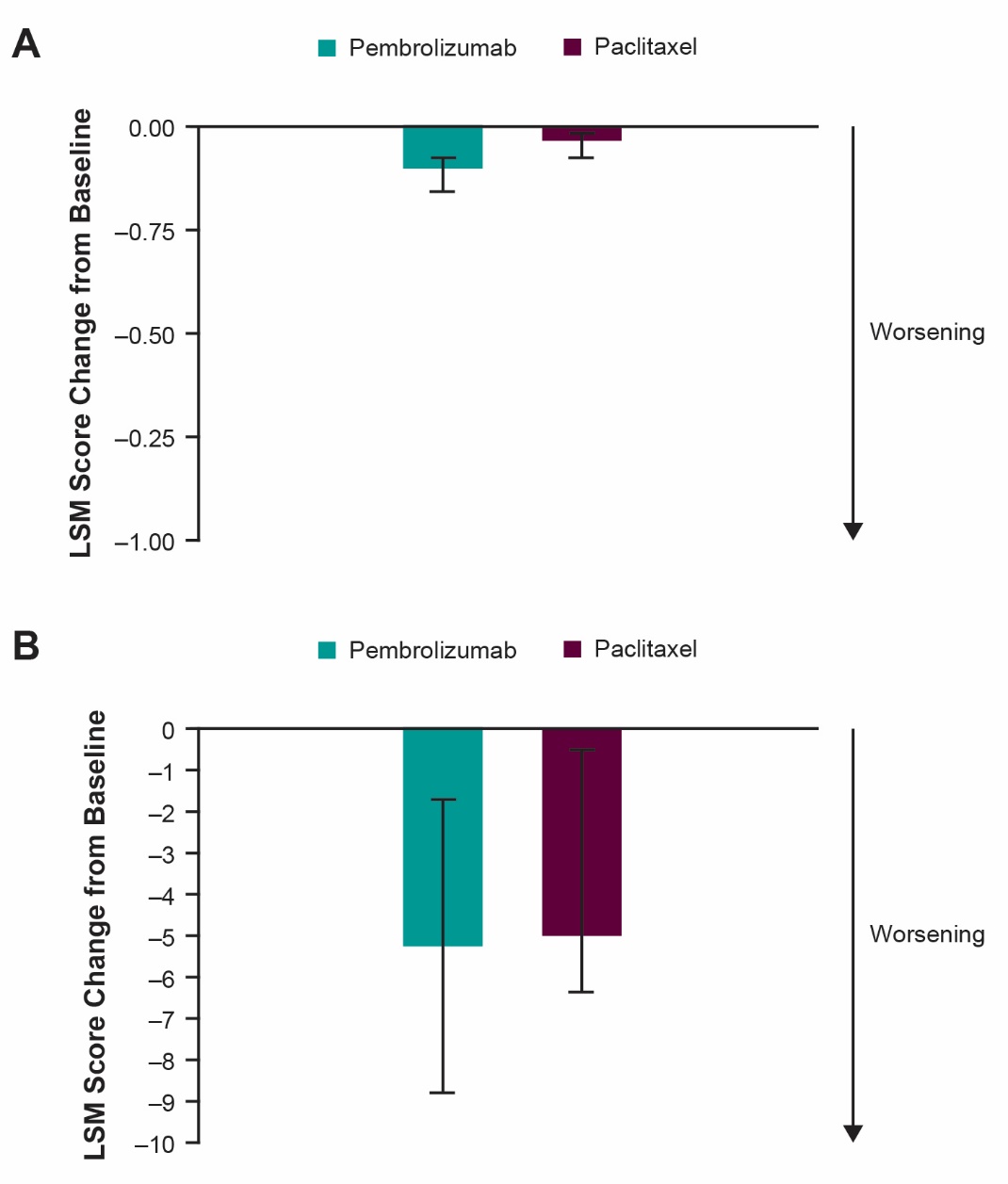

Supplement: Supplementary file 1 — Supplementary file1 (DOCX 115 kb) [file 10120_2021_1200_MOESM1_ESM.docx]
